# Targeting smoking cessation to high prevalence communities: outcomes from a pilot intervention for gay men

**DOI:** 10.1186/1471-2458-4-43

**Published:** 2004-09-30

**Authors:** Richard Harding, James Bensley, Nick Corrigan

**Affiliations:** 1 Department of Palliative Care & Policy, Guy's King's & St Thomas' School of Medicine, King's College London, Weston Education Centre, Cutcombe Road, London, SE5 9PJ, UK; 2 GMFA, Unit 42 Eurolink Centre, 49 Effra Road, London, SW2 1BZ, UK

## Abstract

**Background:**

Cigarette smoking prevalence among gay men is twice that of population levels. A pilot community-level intervention was developed and evaluated aiming to meet UK Government cessation and cancer prevention targets.

**Methods:**

Four 7-week withdrawal-oriented treatment groups combined nicotine replacement therapy with peer support. Self-report and carbon monoxide register data were collected at baseline and 7 weeks. N = 98 gay men were recruited through community newspapers and organisations in London UK.

**Results:**

At 7 weeks, n = 44 (76%) were confirmed as quit using standard UK Government National Health Service monitoring forms. In multivariate analysis the single significant baseline variable associated with cessation was previous number of attempts at quitting (OR 1.48, p = 0.04).

**Conclusions:**

This tailored community-level intervention successfully recruited a high-prevalence group, and the outcome data compares very favourably to national monitoring data (which reports an average of 53% success). Implications for national targeted services are considered.

## Background

Analysis of tobacco marketing has demonstrated lesbian and gay youth as an emerging target community [1], thereby reinforcing behaviour patterns that contribute to the adult gay smoking prevalence (39.7–47.8%) being up to twice that of adult heterosexuals [2].

Gay men have been disproportionately affected by HIV/AIDS disease in developed countries. HIV risk-taking behaviour is associated with cigarette smoking among HIV-negative gay men [3], and among gay men infected with HIV, studies of co-morbidity and survival have identified cigarette smoking as a significant risk factor for opportunistic infections [4] and rapid disease progression [5]. A review of the evidence on sexuality and cigarette smoking found the elevated rates of tobacco use to be consistent across international studies, and concluded with a strengthened call for targeted cessation interventions to lesbians, gays and bisexuals [6]. None have been published to date.

The National Health Service (NHS) Cancer Plan, a UK Government health strategy, recommends that Primary Care Trusts (PCTs) take a commissioning lead in forming local alliances involving community groups, harnessing community efforts, and dissemination of effective interventions. [7]. In order to meet smoking cessation targets, PCTs are recommended to develop specialist smoking cessation services and develop links with local community groups [8].

This innovative pilot study aimed to design, recruit to, and deliver a series of pilot smoking cessation group interventions and to evaluate outcomes using standard UK Government assessment criteria.

## Methods

### Intervention design

The intervention was developed and delivered by a community-based volunteer-led charity in London UK, with a remit to promote the health of gay men. Potential acceptability and effectiveness were maximised by providing an NHS-approved programme adapted for an appropriate service wholly facilitated and attended by gay men. Seven volunteers experienced in delivering group interventions within the organisation were trained in the 3 day course "*Setting up and running specialist Smoking Cessation Clinics"*, part of the Smoking Cessation Training and Research Programme (SCTRP) at St Bartholomew's and Royal London School of Medicine and St George's Hospital Medical School. The programme of withdrawal-oriented treatment combines groupwork, nicotine replacement therapy (obtained on prescription from general practitioners) and ongoing peer support throughout. An initial information session is followed by 6 closed group sessions, setting a quit data for week 3. This pilot consisted of 4 delivered groups, and each group consisted of 7 closed weekly meetings each of 2 hours.

The service principle was for a non-judgemental environment where gay men could address socialising and gay social spaces, recreational drug use, sexuality and HIV and the impact of these on their motivations, and ability, to quit smoking. Several specific modifications were made to the taught model. Our intervention modified the SCTRP program's use of "Quit buddies" which promoted partnered support, instead creating "Quit cells" of 3 or 4 participants. This design modification was made in the light of other group interventions delivered by this community organisation in which reliance of a participant on more than one person for support has found to be more reliable. The information on Zyban was expanded to address contraindications with HIV antiretroviral combination therapies. Exercises from assertiveness training courses were imported to assist participants in clearly communicating the intention to remain a non-smoker. In general, group discussion and processes were focussed on culturally-specific contexts to gay men. A detailed intervention programme was written in order to promote consistency across the cycles of intervention delivery.

Week 1: information on the course content as well as expectations of quit date are given along with information regarding potential side effects and how to deal with them. Week 2: what to expect when you quit and how to deal with reactions, information on the effects of carbon monoxide, preparation for quit date, personal action plan and the how to use a smoking diary. Week 3: information on how to use nicotine replacements, role play of assertive refusal of cigarettes, selection and formation of quit support cells, and personal statements of cessation. Week 4: group review of challenges of the first week of not smoking with reference to smoking diary and personal action plan, exploration of potential "alternative" support such as meditation and exercise, discussion of the challenges of drug use with respect to smoking cessation. Week 5: group review of previous week's experience, information on health benefits achieved to date and weight gain issues. Week 6: review of previous week's experience, identification of future sources of support. Week 7: review of previous week's experience, information of health benefits to date, elaboration on support sources, small celebration of the group's achievement.

### Recruitment

Twenty-four recruitment advertisements were placed in free London-wide and national gay press, and accompanying editorial and articles were secured to support the recruitment process.

### Data collection and analysis

Prior to the initial session, participants were sent the required UK Department of Health self-completion Smoking Cessation Service NHS Client Assessment Form. Carbon monoxide readings were taken at each session from week 2, using the "Smokealyser" calibrated carbon monoxide register, and readings were used in addition to self-report data to confirm smoking cessation at week 7. All intervention attendees were asked to give written permission for data collection purposes and were given guarantees of confidentiality.

All data were entered into SPSS for windows V11. In line with NHS monitoring data requirements, the percentage of successful quitters was calculated as those who gave carbon monoxide readings and confirmed they had quit at week 7 as a percentage of those who set a quit date for week 3. Variables were entered individually into univariate binary logistic regressions, with cessation outcome as the dependent variable and participant baseline characteristics, attitudes and behaviour, and nicotine replacement methods as independent variables. Variables with p values below 0.25 were then entered stepwise into a multivariate logistic regression, with 95% confidence intervals (95% CI) reported.

## Results

### Participant characteristics

Ninety-eight men registered to attend the intervention, and of these 76 attended at least the first session. Sixty-nine of men returned the assessment sheet, and the outcome analysis is of those 69 men.

The mean age of participants was 37.1 years (range 23–63, SD = 7.2 years), and n = 63 (90%) reported their ethnicity as White. Forty-four men (64%) had been educated to degree level or higher, and n = 52 (75%) were in full time employment with a further 9 (13%) men medically retired, n = 5 (7%) unemployed, n = 2 (3%) in full time education and n = 1 (1%) retired. Seventeen men (25%) were entitled to free prescriptions (i.e. the welfare state pays for their prescribed medications). Sixty-five men (94%) reported that they drink alcohol, consuming a mean of 22.8 units per week (median = 20, SD = 19, range 1–120).

### Smoking behaviours at baseline

The daily number of cigarettes smoked was as follows: 1–5 (n = 3, 4%); 5–10 (n = 5, 7%); 11–20 (n = 27, 39%); 21–30 (n = 21, 30%); 31–40 (n = 8, 12%); 41+ (n = 5, 7%). The first cigarette after waking was smoked during the following number of minutes after waking: 5 minutes (n = 19, 28%); 6–30 minutes (n = 31, 45%); 31–60 minutes (n = 7, 10%); 61+ minutes (n = 11, 16%). Smoking motivations are summarised in Table 1.

**Table 1:** Smoking behaviour at baseline

	(Strongly) agree	Neither	(Strongly) Disagree
I enjoy smoking	44 (64)	11 (16)	13 (18)
Smoking helps me cope with stress	38 (55)	15 (22)	15 (21)
Smoking helps me to socialise	39 (57)	16 (23)	13 (19)
Smoking helps me to cope with boredom	31 (45)	22 (32)	15 (21)
I smoke to keep my weight down	5 (7)	7 (10)	55 (80)

### Health status and consultations

Participants reported a mean 2.6 of consultations with their primary care General Practitioner (GP) in the previous year (median = 2, SD 3.5). Secondary/hospital consultations in the previous year were reported by n = 35 (52%) men, with a mean of 2.26 consultations for these men (median = 1, SD = 3.9). Thirty-four men (51%) had been recommended by their GP to give up cigarette smoking, and n = 26 (38%) men were currently on prescribed medication. Fourteen men (20%) were diagnosed HIV-positive, n = 25 (51%) HIV-negative, n = 16 (23%) untested and n = 4 (6%) refused to answer. The participants rated their health as follows: excellent n = 10 (14.5%); good n = 36 (52%); moderate n = 20 (29%); poor n = 2 (3%); very poor n = 1 (1%).

### Quitting motivations and history

Sixty-one men (90%) had made a previous attempt to quit, and of those who had made an attempt the mean was 2.85 attempts (median 3, SD = 1.4). Previously employed nicotine replacement methods were gum n = 30 (49%), patches n = 30 (49%), nasal spray n = 3 (5%), inhalor n = 12 (20%), microtabs n = 3 (5%), nicotine lozenges n = 4 (7%), and Bupropion (Zyban) n = 12 (20%).

Participants described the importance of this current attempt to quit as extremely important (n = 33, 48%); very important (n = 27, 39%); quite important (n = 9, 13%); not at all important (n = 0). Participants rated their chances of quitting for good on this attempt as extremely high (n = 10, 15%); very high (n = 27, 39%); quite high (n = 24, 35%); not very high (n = 7, 10%); very low (n = 1, 1%).

### Intervention attendance and outcomes

Attendance at sessions was consistently high, of 532 person-sessions 13 sessions were missed. Non-attendance did not apparently cluster around a particular session.

At week 3, of the 69 men who gave data, n = 58 men (84%) set a quit date. At week 7 (4 weeks after the quit date) n = 44 men (64%) were confirmed as having quit using the CO monitor, representing 58% of those who attended the first session, 76% of those who set a quit date and 64% of those who gave data at baseline and week 7.

A further 3 men reported by telephone that they had quit smoking but did not attend the final session to give clinical data to verify. Nine men (13%) reported not having stopped smoking, n = 6 men (9%) set a quit date at week 3 and did not return to group, n = 7 men (10%) attended the first session only. For the purposes of this analysis, these 25 men were coded as not having quit in the following modelling.

### Variables associated with cessation outcomes in multivariate logistic regression

This analysis considers those 44 men confirmed as having ceased compared to those 25 categorised as not having quit. Following univariate analysis (see Table 2), the 4 variables entered into the multivariate model were smoking for enjoyment, number of cigarettes per day, smoking to keep weight down, and number of previous attempts. Only the latter (continuous) variable was significantly associated with successful quitting at week 7 (OR = 1.48, 95% CI = 1.02, 2.14, p = 0.04). Data from the multivariate model are presented in Table 3.

**Table 2:** Univariate binary regression analysis of demographic and behavioural baseline data with respect to cessation outcomes

Variable	p	Odds Ratio	95% CI
Age	0.36	1.03	0.96, 1.11
"I enjoy smoking"	0.22*	1.38	0.82, 2.32
"Smoke helps me cope with stress"	0.39	0.82	0.53, 1.29
"Smoking helps me to socialise"	0.58	0.89	0.58, 1.36
"Smoking helps me to cope with boredom"	0.97	0.99	0.63, 1.55
"I Smoke to keep down weight"	0.18*	1.41	0.85, 2.33
No. of cigarettes per day	0.08*	0.67	0.42, 1.05
Time to 1^st ^daily cigarette	0.52	1.18	0.71, 1.96
No. of previous attempts	0.04*	1.44	1.02, 2.01
Importance of this attempt	0.80	0.91	0.45, 1.84
No. of GP visits	0.33	1.09	0.92, 1.29
No. of secondary care visits	0.43	1.06	0.92, 1.22
Expected chance of quitting success on this attempt	0.91	0.97	0.58, 1.62
Perceived health status	0.96	1.02	0.55, 1.90

* Included in multivariate analysis (see Table 3)

**Table 3:** Multivariate analysis of variables identified as associated with cessation at week 7 (i.e. p < 0.25 in univariate analysis, Table 2).

Variable	p	Odds Ratio	95% CI
No of cigarettes per day	0.15	0.68	0.41, 1.14
Smoking to keep down weight	0.20	1.43	0.83, 2.45
No of previous attempts to give up	0.04*	1.48	1.02, 2.14
I enjoy smoking	0.38	1.30	0.73, 2.30

## Discussion

This pilot intervention has targeted a hitherto overlooked high smoking prevalence group, and has adapted a Government-approved intervention to meet the specific needs of gay men in an appropriate and acceptable setting. The success rate of 76% of men who had set a quit date being confirmed as having quit at week 7 compares extremely favourably to national monitoring data, which reports a success rate nationally 2001–2002 for smoking cessation services as 53% [9].

Public health targets must consider the needs of high prevalence communities, and this may be achieved through innovative development of existing effective services. However, this study has highlighted the lack of targeted interventions for gay men, and the evidence demonstrates further elevated health needs compared to the general population in the fields of alcohol and drug use [10] mental health [11] and cancer [12,13].

Further research may identify the factors which contributed to the effectiveness of this pilot complex participative intervention, including offering recruitment and delivery outside of community settings, measuring success rates for gay men in non-gay specific or tailored groups, and the usefulness of "quit cells". Longer-term follow-up data and increasing dosage to include a follow-up session would also provide further useful data. In order to refine the intervention for trial testing, qualitative data regarding the utility, acceptability and preferences for the content of specific sessions would be illuminating. Further, the non-randomised design without comparison group limits presents a limitation to the generalisability of findings, yet still offers cessation outcomes much better than standard national cessation data quoted above which were collected without quasi-experimental design using the same follow-up period. Data were not available on the 29 men who registered for the course but did not attend or complete baseline data, and so it is not possible to compare their demographics or smoking behaviours to those who took up the intervention. Certainly, replication of this first pilot would be necessary in other settings, e.g. non-metropolitan communities, where issues of feasibility and uptake should be addressed. Commissioners may consider the purchase of existing facilitators from cities to deliver in non-metropolitan areas where demand is likely to be lower, as smoking cessation service recommendations state that group leaders need to keep up to date with their skills and to use them on a regular basis [14].

## Conclusions

In order to meet the smoking cessation needs of this hitherto overlooked population, and to meet public health policy targets, a rigorous research agenda must be established. While the use of required standard outcome monitoring must be continued, rigorous experimental trials using longer term follow up and commonly reported measures are required. Complex participative interventions must be developed, as in this pilot, from evidence-based interventions with full programme description to ensure replication. The development of appropriate interventions must first pilot services to ensure that they are appropriately adapted to maximise acceptability and uptake among target communities.

Lastly, provision of the service by skilled volunteer facilitators has ensured an acceptable, low-cost intervention with a rate of effectiveness in these four pilot groups that compares favourably to national non-targeted interventions outcomes calculated using standard assessment formula. Acceptability of the model appears high with respect to the low number of missed sessions. Voluntary sector provision and delivery should be considered as a low-cost and highly acceptable point of delivery for effective community-level smoking cessation interventions.

## Competing interests

The authors declare that they have no competing interests.

## Authors' contributions

JB and NH managed the pilot study. RH was responsible for data management and analysis and drafted the manuscript. All authors read and approved the final manuscript.

## Pre-publication history

The pre-publication history for this paper can be accessed here:


